# Using the R = MC^2^ heuristic to assess whole-of-school physical activity implementation in elementary schools: a cross-sectional study

**DOI:** 10.1186/s12966-025-01815-7

**Published:** 2025-08-23

**Authors:** Derek W. Craig, Kevin Lanza, Christopher D. Pfledderer, Andjelka Pavlovic, Kempson Onadeko, Natalia I. Heredia, Jizyah Injil, Laura F. DeFina, Timothy J. Walker

**Affiliations:** 1 Department of Health Promotion and Behavioral Sciences, UTHealth Houston School of Public Health, Houston, Texas, USA; 2 Institute for Implementation Science, UTHealth Houston, Houston, Texas, USA; 3 Department of Environmental and Occupational Health Sciences, UTHealth Houston School of Public Health, Austin, Texas, USA; 4 Department of Health Promotion and Behavioral Sciences, UTHealth Houston School of Public Health, Austin, Texas, USA; 5 Kenneth H. Cooper Institute at Texas Tech University Health Sciences Center, Dallas, Texas, USA

**Keywords:** Schools, Physical activity, Implementation, Organizational readiness

## Abstract

**Background:**

Schools are recommended to use a whole-of-school (WOS) approach to promote physical activity opportunities before, during, and after school. Yet, the barriers and facilitators to implementing a WOS approach successfully are not well understood. The R = MC^2^ heuristic, which defines readiness for implementation as a combination of an organization’s motivation and capacity to implement, can enhance our understanding of implementation in the school setting. This study examines associations between constructs from the R = MC^2^ heuristic and schools’ implementation of a WOS approach.

**Methods:**

We conducted a secondary analysis of cross-sectional data from U.S. elementary schools participating in the NFL PLAY60 FitnessGram Project during the 2022–23 school year. From surveys administered to school staff, we created a WOS index (range = 0–12) comprising six physical activity practices: physical education, recess, before and after-school programs, classroom-based approaches, and active transport. We also assessed how six constructs from the R = MC^2^ heuristic (i.e., culture, implementation climate, leadership, priority, resources utilization, resource availability) impact physical activity implementation using a series of questions measured on a 5-point Likert scale. We used linear regression models to determine associations between R = MC^2^ constructs (independent variables) and WOS index scores (dependent variable), controlling for school-level characteristics (student enrollment, percentage of race/ethnicity and economically disadvantaged students served) and state-level clustering.

**Results:**

The analytic sample consisted of 132 schools across 18 states. On average, school staff rated leadership (mean = 4.1, range = 1.5–5) and organizational culture (mean = 4.0, range = 2.25–5) the highest. The mean WOS index score was 6.1. Partially adjusted models indicated significant positive associations between each R = MC^2^ construct and WOS index scores. Fully adjusted regression models revealed priority (*b* = 0.88; *p* = 0.010; 95% CI = 0.19–1.56) and implementation climate (*b* = 0.69; *p* = 0.047; 95% CI = 0.07–1.32) were positively and significantly associated with WOS index scores.

**Conclusions:**

Our study provides insights into key implementation constructs associated with providing school-based physical activity opportunities. These findings can support the development of resources and implementation strategies which, in turn, can help schools address implementation-related disparities. This will help schools improve the quality and accessibility of opportunities for physical activity provided to students across the United States.

## Background

Most youth in the United States (U.S.) are insufficiently active despite the well-documented health benefits associated with physical activity [1]. One way to increase youth physical activity levels is through school-based promotion. Schools reach nearly all children in the U.S. and play an important role in supporting student health [2]. Authorities recommend schools promote physical activity through a Whole-of-School (WOS) approach wherein opportunities for physical activity are integrated into each segment of the school day: before (e.g., before-school programs, active transportation), during (e.g., physical education, recess, classroom-based approaches), and after (e.g., after-school programs, active transportation) [3]. Successful implementation of a WOS approach is dependent on a range of school stakeholders (e.g., leadership, teachers, parents) and the school environment to ensure opportunities provided are accessible and inclusive to all students.

Past research has identified numerous challenges to promoting physical activity in the school setting [4]. Common school-level barriers to implementation include competing priorities, limited time and resources, lack of leadership support, and insufficient staff buy-in [4–7]. Other contextual factors that influence what and how physical activity is provided include the geographic location of the school, the percentage of economically disadvantaged students served, and the racial/ethnic makeup of the school’s student population [8–13]. However, the school context can look very different from one to the next. Therefore, assessing these contextual factors is critical to informing implementation strategy development and improving the reach and effectiveness of physical activity opportunities.

The field of implementation science offers methods to systematically identify the contextual factors underlying the inconsistent delivery of evidence-based programs [14]. Theories and frameworks from implementation science often focus on specific phases of implementation (e.g., adoption, implementation, maintenance) thus making it easier to understand and explain how and why implementation efforts succeed or fail [15]. One specific concept relevant to implementation is organizational readiness. The R = MC^2^ heuristic (*r*eadiness = *m*otivation x innovation-specific capacity x general *c*apacity) defines readiness as a combination of an organization’s motivation and capacity and is comprised of constructs such as leadership, culture, and priority known to be associated with successful implementation [16]. Organizational readiness, however, is poorly understood in the school setting [17]. Measuring constructs from the R = MC^2^ heuristic can help schools and educators identify and mitigate potential barriers [18, 19] and inform the design of tailored implementation supports specific to a school’s needs.

Improving our understanding of school-level contextual factors will help enhance the implementation of the whole-of-school approach to physical activity within the school setting. The R = MC^2^ heuristic can provide guidance on the selection of factors to assess but few published studies applying the framework to school-based physical activity exist [5, 20]. More investigation is needed to determine the contextual factors influencing implementation readiness as it pertains to school-based physical activity promotion. Therefore, we examined the relation between constructs (from the R = MC^2^ heuristic) and the extent to which a sample of elementary schools geographically dispersed across the U.S. provide physical activity opportunities consistent with a WOS approach. The findings can support the development of implementation strategies designed to improve the adoption and implementation of WOS physical activity opportunities. This will improve the physical activity opportunities provided by schools and increase physical activity levels among youth in the U.S.

## Methods

### Participants, study design, and setting

We conducted a secondary analysis of cross-sectional survey data collected from elementary schools participating in the NFL PLAY 60 FitnessGram Project during the 2022–2023 school year. The NFL PLAY 60 FitnessGram Project is a collective impact initiative of The Cooper Institute and funded by the National Football League (NFL) Foundation. The program is designed to improve the health and well-being of youth and operates within the 32 NFL markets and surrounding areas. An NFL PLAY 60 steering committee selects schools to participate based on their proximity to and alignment with the priorities of the NFL club, as well as their health and financial needs. Participating schools receive multiple incentives (e.g., NFL flag kits, fitness equipment, and FitnessGram kits) and resources (e.g., PLAY 60 App, EVERFI learning modules, and physical activity promotion materials) that teachers and staff can use to improve current physical activity offerings (e.g., PE, afterschool programs), promote student’s health and well-being, and foster a healthier school environment. Approximately 160 new schools are onboarded into the program annually and are able to continue participating in the program each year based on the availability of funding.

As part of the NFL PLAY 60 FitnessGram Project, each participating school designates a representative, most commonly a PE teacher, to complete a one-time initiation survey at enrollment and an end-of-year survey annually. Herein, we focused on the end-of-year survey data collected during the Spring 2023 semester from a convenience sample of elementary schools geographically dispersed across the United States. The Cooper Institute staff sent surveys to 220 elementary schools electronically via Qualtrics. The end-of-year survey assesses NFL PLAY 60 programming, school-based physical activity opportunities offered throughout the year, and the barriers and facilitators to implementation. All survey respondents consented prior to completing the survey. This study was approved by the Institutional Ethics Review Board at The Cooper Institute and the Committee for the Protection of Human Subjects at the University of Texas Health Science Center at Houston School of Public Health (HSC-SPH-23-0098).

### Implementation variables

We assessed six constructs from the R = MC^2^ heuristic: available resources, implementation climate, leadership, organizational culture, priority, and resource utilization (Table 1). We selected these constructs based on their theoretical relevance to school-based implementation and evidence from the physical activity literature [21]. The items used to measure each of the six implementation constructs were adapted from measures with strong psychometric properties to best-fit the constructs [22, 23]. Adaptations included superficial wording changes and reducing the number of items used to assess a construct. We assessed each construct using between 2 and 4 items. Participants rated their level of agreement with each statement using a 5-point Likert scale (1-strongly disagree to 5-strongly agree). We aggregated the responses and used the mean score of the scale items.

**Table 1:** R = MC^2^ constructs, definitions, and number of survey items

Construct	Definition	Number of Survey Items
Available resources	The level of resources dedicated to implementation and ongoing operations, including money, training, education, physical space, and time	4
Implementation climate	The absorptive capacity for change, shared receptivity of involved individuals to an intervention, and the extent to which the use of that intervention will be rewarded, supported, and expected within the organization	3
Leadership	Whether power authorities within the school articulate and support organizational activities	4
Organizational culture	Norms, values, and basic assumptions of a given organization	4
Priority	Individuals’ shared perception of the importance of the implementation within the organization	2
Resource utilization	How discretionary/uncommitted resources are devoted to innovations	4

### Whole-of-School physical activity variables

The end-of-year NFL PLAY 60 FitnessGram survey contains questions about each of the six components of the WOS approach: (1) PE, (2) recess, (3) before- and (4) after-school physical activity programs, (5) classroom-based physical activity approaches, and (6) active transportation (see Supplemental File). For PE and recess, respondents answered a series of questions used to create a weekly minutes variable for each respective component. For PE, respondents reported the number of days per week PE was provided to students and the duration of each PE class. The survey employed ordered categorical response options (e.g., 20–25 min, 26–30 min) for the duration question; therefore, we used the median categorical value in the calculations for weekly minutes (e.g., 22.5 min). Additionally, we calculated an average for weekly minutes of PE for schools using an alternating PE schedule (e.g., 1 day of PE one week, 2 days of PE another). We then categorized PE values into three groups: 0 (low): < 75 min per week, 1 (medium): 75–149 min per week, and 2 (high): ≥ 150 min per week. For recess, respondents reported the number of times per day, days per week, and minutes per session recess was offered. We then categorized recess values using a similar three-group approach: 0 (low): < 100 min per week, 1 (medium): 100–149 min per week, and 2 (high): ≥ 150 min per week. Groupings for PE and recess were informed by the sample distributions and recommendations for daily PE and recess put forth by SHAPE America and the U.S. Centers for Disease Control and Prevention [24–27]. 

For the remaining WOS components (before- and after-school programs, classroom-based physical activity approaches, and active transportation), respondents answered items measured on a 5-point Likert scale. Respondents answered two separate questions about the accessibility of before- and after-school physical activity programming. One survey question asked respondents to report on the use of classroom-based physical activity approaches within their school. Two additional questions assessed the encouragement of and accessibility to active transportation opportunities. For classroom-based physical activity approaches and before- and after-school programs, the numerical value from the 5-point Likert scale corresponding to the respondent’s answer choice was assigned as the component score (e.g., strongly disagree = 1). For active transportation, a mean value from the two questions was calculated. We categorized schools as low (< 3), medium (3–3.9), or high (≥ 4) based on their responses. These cutoffs align with the 5-point Likert scale, where scores ≤ 3 indicate disagreement with the question, a score of 3 reflects neutrality, and scores of ≥ 4 represent agreement with the question.

Similar to previous studies, we created a WOS index variable by summing the scores across each of the six WOS components [8, 28]. Individual WOS components could contribute up to two points (0 - low, 1 - medium, or 2 - high), allowing the WOS index values to range from 0 to 12. We assigned a missing value to the overall WOS index variable if any questions from the survey pertaining to the WOS components were unanswered by respondents.

### School characteristics variables

We also obtained publicly available school characteristics data from the National Center for Education Statistics (NCES) [2]. We downloaded data for the following variables: school locale (city, suburban, town/rural), the number of students served by race/ethnicity (i.e., school composition), the number of economically disadvantaged students served, and total student enrollment. The number of students by race/ethnicity (as reported by NCES) was divided by the total student enrollment to create a school-level variable that represents the student body served by each school. We categorized each school into one of five groups: majority non-Hispanic White (> 50%), majority non-Hispanic Black (> 50%), majority Hispanic (> 50%), majority another race/ethnicity (e.g., American Indian, Asian, two or more races) (> 50%), and diverse (no single race/ethnicity group > 50%). Additionally, the economically disadvantaged students served variable was informed by the number of students within a school who are eligible for free or reduced-price lunch. This number was converted into a percentage by dividing the number of economically disadvantaged students by the total number of students enrolled at the school.

### Statistical analysis

Prior to analysis, we screened the data to assess survey completion and distributions of the independent variables (implementation constructs) and dependent variables (WOS index). This led to the removal of 14 duplicate responses, 11 unrealistic responses (e.g., 7 days/week of PE), and 8 incomplete responses (e.g., completed < 10% of the survey). Next, we ran a series of linear regression models to examine associations between R = MC^2^ constructs and the WOS index. Partially adjusted models included each R = MC^2^ construct entered individually and adjusted for school characteristic variables. We then examined a fully adjusted model that included all R = MC^2^ variables and adjusted for school characteristic variables. School characteristic variables included total student enrollment, percentage of economically disadvantaged students served, percentage of students served by race/ethnicity, NFL PLAY 60 cohort, and state-level clustering. We used StataSE 18 to complete the analyses and indicated statistical significance using a p-value of < 0.05.

## Results

The final analytic sample consisted of 132 elementary schools across 18 states (Table 2). About 58% (*n* = 76) of schools were located within cities. NCES data indicated the percentage of economically disadvantaged students served by schools in the sample ranged from 2.2 to 100%. The percentage of students served by race/ethnicity was relatively balanced with schools characterized as diverse (31.1%, *n* = 41) and Hispanic (30.3%, *n* = 40) making up over half of the sample followed by schools serving mostly non-Hispanic White (20.5%, *n* = 27) and non-Hispanic Black students (15.9%, *n* = 21).

**Table 2:** School-level demographic information

Variable	School Sample (*n* = 132)
Student enrollment (*M*, SD)	457.4 (201.2)
Percentage of economically disadvantaged students (*M*, SD)	68.2 (28.2)
Eligible for Title I (n, %)	101 (76.5)
Geographic location (n, %)	
City	76 (57.6)
Suburban	43 (32.6)
Rural/town	13 (9.9)
School race/ethnicity (n, %)	
Majority non-Hispanic Black (≥ 50%)	21 (15.9)
Majority Hispanic (≥ 50%)	40 (30.3)
Majority non-Hispanic White (≥ 50%)	27 (20.5)
Majority another race/ethnicity (≥ 50%)	3 (2.3)
Diverse (no single racial/ethnic group ≥ 50%)	41 (31.1)

Note: Six schools were missing Title 1 data

On average, school staff rated leadership (mean = 4.1; SD = 0.8) and organizational culture (mean = 4.0; SD = 0.7) the highest. In contrast, implementation climate (mean = 3.5; SD = 0.9) and resource availability (mean = 3.7; SD = 0.9) were rated the lowest. The full range of the scale was used by respondents for each of the six constructs although the distributions of the composite scores, in general, were negatively skewed (Table 3).

**Table 3:** Descriptive information for key study variables

Variable	Mean (SD)
*R = MC*^*2*^ *constructs*	
Available resources	3.7 (0.9)
Implementation climate	3.5 (0.9)
Leadership	4.1 (0.8)
Organizational culture	4.0 (0.7)
Priority	4.0 (0.8)
Resource utilization	4.0 (0.8)
*Whole-of-school components*	
Physical education minutes per week	78.0 (45.0)
Recess minutes per week	139.0 (76.1)
Before-school programming	2.5 (1.6)
After-school programming	3.7 (1.4)
Classroom-based physical activity	2.9 (1.0)
Active transportation	3.3 (1.0)
Whole-of-school index	6.1 (2.3)

Note: Active transportation, classroom-based physical activity, and before- and after-school programming were measured using a 5-point scale

The mean WOS index value was 6.1 (SD = 2.3). Scores on the WOS index ranged from 1 to 11 and were normally distributed. Schools reported offering 2 days of PE per week, on average, for a total of about 79 min per week, whereas recess was offered almost daily (mean = 4.7 days) for a total of about 140 min per week. Classroom-based physical activity approaches were reportedly used by about half the teachers and staff at participating NFL PLAY 60 schools. In addition, respondents indicated after-school physical activity programs were more accessible to students than physical activity programs occurring before school. Assessing trends across schools indicated students were encouraged to walk or bike to school; however, schools inconsistently offered accessible active transport programming.

Partially adjusted linear regression models revealed significant positive associations between all six implementation constructs and the WOS index (Table 4). After adjusting for covariates and state-level clustering, priority (*b* = 0.89; *p* = 0.02; 95% CI = 0.17–1.61) and implementation climate (*b* = 0.73; *p* = 0.03; 95% CI = 0.07–1.40) remained significantly associated with the WOS index in the full model. There were no significant associations found for available resources, leadership, organizational culture, and resource utilization.

**Table 4:** Results from linear regression models

	WOS Index	
Model Variables (*n* = 132)	Partially adjusted^a^ *b* (95% CI)	Fully adjusted^b^ *b* (95% CI)
Available resources	**0.73 (0.19–1.27)**	−0.21 (−0.80-0.38)
Implementation climate	1.14 (0.82–1.46)**	**0.73 (0.07–1.40)**
Leadership	1.27 (0.87–1.67)**	0.21 (−0.78-1.20)
Organizational culture	1.29 (0.69–1.89)**	0.45 (−0.42-1.31)
Priority	1.37 (0.86–1.87)**	**0.89 (0.17–1.61)**
Resource utilization	1.06 (0.59–1.55)**	−0.27 (−1.14-0.60)

Note: Boldface indicates statistical significance of *p* < 0.05. *p < 0.01; **p < 0.001

^a^Partially adjusted models included one construct and adjusted for school characteristic variables

^b^Fully adjusted models entered all constructs and adjusted for school characteristic variables

## Discussion

This study assessed the relation between implementation constructs from the R = MC^2^ heuristic and the extent to which elementary schools geographically dispersed across the U.S. provide physical activity opportunities consistent with a WOS approach. Schools reporting greater prioritization of school-based physical activity opportunities were found to have higher WOS index scores. A supportive implementation climate was also found to be associated with WOS implementation. These findings suggest improving the R = MC^2^ constructs may be a way to improve how physical activity opportunities are provided to students. Developing strategies to ensure school leadership, teachers, and staff understand the importance of physical activity and a supportive environment may be most helpful in enhancing school-based physical activity promotion.

Our findings indicate prioritizing physical activity was positively associated with the WOS index. This finding is notable given physical activity often faces competing priorities in schools. For example, time constraints resulting from a crowded curriculum and an emphasis on increasing student’s academic performance have commonly been cited as educational priorities that prevent the provision of physical activity during the school day [6, 29, 30]. Importantly, these barriers are not experienced equally across schools. Schools serving predominantly racially and ethnically minoritized students or located in economically disadvantaged communities often face greater implementation challenges due to limited resources [9, 31, 32], high staff turnover [33, 34], and reduced access to supportive infrastructure [34–36]. These inequities can make it especially difficult to prioritize and sustain physical activity initiatives. Therefore, as part of the NFL PLAY 60 FitnessGram project, schools are provided with resources, equipment, and incentives to improve the quality of school-based physical activity opportunities. These types of supports, which are critical to implementation [37], likely motivate schools to place greater priority on physical activity and may help to explain our findings. Schools looking to further increase the perceived priority placed on physical activity should focus on communicating the benefits of physical activity to teachers and staff and establishing goals related to physical activity promotion [7, 38, 39]. Other practical strategies include adding agenda items to school-wide staff meetings to facilitate discussion on relevant physical activity topics and creating systems to monitor the implementation of physical activity opportunities [40]. 

Research has shown implementation climate is critical to the success of school-based physical activity opportunities. [21] Yet, few studies have quantitatively examined how implementation climate is associated with providing physical activity opportunities in the school setting. Similar to the present study, both Craig et al. [21] and Carlson et al. [41] found a more supportive implementation climate was associated with greater implementation success. Our study also provides additional evidence of the importance of implementation climate given that the previous studies involved much fewer schools (24 and 46 schools, respectively). Higher ratings on implementation climate may be attributed to the programming support (e.g., training, technical assistance, webinars) provided to teachers and staff as well as the guidance provided to school leaders for developing an active school environment. To improve (or maintain) implementation climate, school stakeholders (e.g., principals and physical activity champions) should consider different types of positive reinforcements (e.g., incentives and shoutouts at staff meetings) that demonstrate support and increase staff buy-in for providing physical activity during the school day.

Although failing to reach statistical significance (*p* < 0.05) in the fully adjusted models, partially adjusted models revealed significant associations for the remaining implementation constructs. These constructs, particularly leadership and available resources, are known to be associated with successful implementation in the school setting [37, 42–45]. Among the constructs assessed, leadership was rated the highest by survey respondents compared to other constructs. The lack of associations in the final model may be due to social desirability and respondents not wanting to speak poorly of school leadership [46]. Furthermore, the lack of association for available resources may be due to all participating schools being provided with the same resources and materials limiting the variation in survey responses. Additional quantitative studies are needed to examine these constructs further and may benefit from including a comparison sample not receiving ongoing intervention support.

Our study has several limitations that should be considered. First, we assessed a limited number of constructs from the R = MC^2^ heuristic (6 out of 19). These six constructs were supported by existing literature or hypothesized by the research team to be theoretically relevant to the implementation of school-based physical activity opportunities. However, additional studies need to assess the full range of readiness constructs to better understand how they influence implementation in the school setting. We also did not assess how well the WOS components were implemented, which limits our understanding of actual practice. Additionally, the sample of schools included in the study were actively participating in the NFL PLAY 60 FitnessGram project. Although these schools were geographically diverse and located across the U.S., they are likely to be more motivated to provide physical activity opportunities which may limit the generalizability of our findings. The study also used a measure with limited psychometric testing, which may have contributed to additional measurement error in the PE variable due to its ordinal response options. Lastly, the cross-sectional nature of the study limits our ability to draw firm conclusions about causal relationships.

## Conclusions

Our study provides key insights into constructs associated with how schools are providing physical activity opportunities consistent with a WOS approach. Our findings note the importance of prioritizing physical activity within the school and instilling a climate supportive of implementing physical activity opportunities. These findings can support the development of resources and implementation strategies which, in turn, can help schools address implementation-related disparities. This will help schools improve the quality and accessibility of opportunities for physical activity provided to students across the United States.

## Data Availability

Data is provided within the manuscript. The datasets used and/or analyzed during the current study are available from the corresponding author upon reasonable request.

## Abbreviations

NCES: National Center for Education Statistics
NFL: National Football League
PE: Physical education
U.S.: United States
WOS: Whole-of-school
